# Assessing Stress Resilience After Smolt Transportation by Waterborne Cortisol and Feeding Behavior in a Commercial Atlantic Salmon (*Salmo salar*) Grow-Out Recirculating Aquaculture System

**DOI:** 10.3389/fphys.2021.771951

**Published:** 2022-01-27

**Authors:** Erik Höglund, Paulo Fernandes, Paula Rojas-Tirado, Jan Thomas Rundberget, Ole-Kristian Hess-Erga

**Affiliations:** ^1^Norwegian Institute for Water Research (NIVA), Oslo, Norway; ^2^Center of Coastal Research, University of Agder, Kristiansand, Norway

**Keywords:** waterborne cortisol, fish welfare, behavior, close containment rearing, welfare indicators

## Abstract

Sampling protocols and water quality sensors have been developed to assess fish health and welfare in recirculating aquaculture systems (RASs). Still, the use of fish-based non-invasive welfare indicators, reflecting the physiological state of the fish, is limited in this type of system. Cortisol, the major stress-coping hormone in fish, diffuses through the gills. Consequently, waterborne cortisol is a potential fish-based non-invasive welfare indicator in RAS. However, its use in commercial rearing systems is sparse. In this study, we evaluated water cortisol levels and feeding behavior as welfare indicators of newly inserted smolt in a commercial RAS for harvest size Atlantic salmon. The RAS consisted of two parallel fish rearing raceways, raceways 1 and 2, sharing the same water treatment with common outlets and inlets. The smolts were inserted in raceway 1 while salmon that have been in the system for 6 months or more were kept in raceway 2. The smolt insertion period was 3 days. Samples for water cortisol levels were withdrawn the day before, 1 and 3 days after the smolt insertion period. Smolt insertion resulted in elevated water cortisol concentrations in the entire system, with the highest values in raceway 1, one day after smolt insertion. Estimated cortisol production in newly inserted smolt decreased over time, was similar to what has been reported in salmon adapting to experimental tanks. Feeding behavior indicated that the appetite was not fully resumed in the newly inserted smolts, while the appetite of fish in raceway 2 was unaffected by smolt insertion. These results, obtained in a highly intensive commercial RAS, suggest that waterborne cortisol together with feeding behavior can be used as indicators for adaptive processes associated with stress resilience in farmed fish. Thus, they are promising non-invasive indicators for assessing the impact of potential stressors on fish welfare in this type of rearing system.

## Introduction

Generally, recirculating aquaculture systems (RASs) minimize the environmental impact and provide a venue to balance aquaculture growth and environmental/ethical considerations (Martins et al., 2010). However, fish health and welfare issues related to water quality and rearing densities in this type of rearing system have been raised (van de Vis et al., 2020). Accordingly, water quality sampling protocols and sensor technologies have been developed to ensure cost-effective production (Su et al., 2020), supported by maintaining good rearing conditions in terms of water quality and fish health and welfare. In addition to water quality-based health and welfare indicators, techniques for monitoring the physiological state of the fish provide important information needed to safeguard the health and welfare of farmed fish (Baretto et al., 2022). Currently, fish-based non-invasive welfare indicators are often based on behavioral and respiratory changes, and the use of other measures is limited (Baretto et al., 2022).

There are several studies showing that hormone levels in the water reflect the physiological status of fish (Ellis et al., 2004; Scott and Ellis, 2007). Since steroids are quite stable in water and play a central role in stress coping and health of fish, many of the studies on water-borne hormones focus on these types of hormones or their metabolites in the rearing water (reviewed by Scott and Ellis, 2007). Generally, waterborne steroids and their metabolites are directly related to steroid clearance in fish, which in-turn depend on steroid lipophilicity; free steroids (lipid-soluble) diffuse into the water across the gills whereas sulfate and glucuronide steroids (lipid-insoluble steroids) are excreted more slowly through the kidneys (Vermeirssen and Scott, 1996; Ellis et al., 2005). Accordingly, several studies show a strong relationship between blood plasma levels of cortisol, the main stress-coping hormone in fish, and the release of free cortisol to the rearing water [for references see review by Scott and Ellis (2007)]. Following this, waterborne cortisol has been used as a noninvasive indicator of stress in laboratory studies (Scott and Ellis, 2007; Félix et al., 2013) and experimental aquaculture facilities (Mota et al., 2017a,b). Still, its use as a non-invasive fish-based welfare indicator in commercial RAS is limited.

In addition to reflecting the physiological status of the fish, steroids, and their metabolites can induce behavioral and physiological changes in fish by acting as pheromones. Indeed, water-borne steroids and their metabolites are detected with great sensitivity and specificity by the olfactory organs of fish and exert important effects on behavior and physiology in major taxa, such as carps (goldfish), catfishes, salmon, and gobies (Chung-Davidson et al., 2010; Good and Davidson, 2016). Considering that cortisol and other steroids have been shown to accumulate in RAS, concerns about the impact of these water-borne hormones on farmed fish have been raised (Mota et al., 2014, 2017b; Good and Davidson, 2016).

In RAS production of slaughter size salmon, smolts are transferred from an external or internal smolt production site. Such transfer/transport consists of several traumatic perturbations, including capture, loading, transport, unloading, and re-stocking. Thus, transfer/transport can initiate a severe stress response in farmed fish (Barton and Iwama, 1991), including release of the stress hormone cortisol (Iversen et al., 1998, 2005). Generally, this hormone redistributes energy from maintenance functions, including growth and immune reactions, towards processes needed for coping with perturbations (Sapolsky et al., 2000). In accordance with this, mortality during the sea phase of the Atlantic salmon production cycle have been associated with transportation stress during the smolt stage (Iversen et al., 2005). However, information on the impact of smolt transport on stress coping and resilience in the sea phase of the production cycle is sparse. However, information on the impact of smolt transport on stress coping and resilience in the sea phase of the rearing cycle is sparse.

In RAS, the dynamics of waterborne cortisol offers a potential indicator of how smolt transportation affects stress resilience in the seawater phase of salmon rearing. Thus, in this study, we evaluated if water-borne cortisol could be used as an indicator for stress resilience in newly inserted smolt in commercial RAS. In addition, potential physiological, and behavioral effects of being exposed to water from newly inserted smolt were investigated. This was done by measuring water cortisol concentrations before and after smolt insertion at a commercial grow-out salmon RAS. In this system, smolts were inserted in one of the two circular parallel raceways with shared water treatment. Cortisol production rate before and after smolt insertion in the two raceways was estimated. In addition, the potential effects of smolt insertion on feeding behavior were quantified.

## Materials and Methods

### Study Site

**FIGURE 1:**
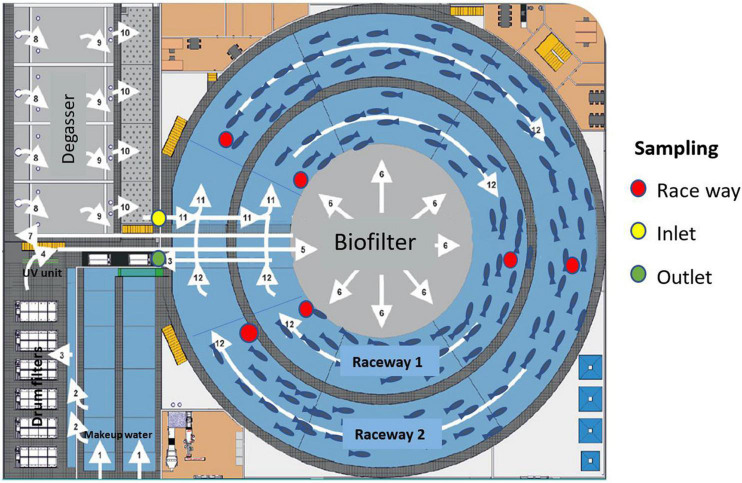
Illustration of a RAS module at Fredrikstad Seafood – water flow through the water treatment loop (white arrows show the direction of flow). The system is comprised of circular raceways for holding fish with a volume of 7,300 m^3^, six drum filters of different mesh sizes (ranging from 20 to 50 μm), a biofilter with five chambers, and a degassing unit. New water (1–2) enters the systems and goes (3) through six drum filters of different mesh sizes (20–50 μm). The permeate from the drum filters is split (10%) into a UV contact chamber (4) and (90%) in a 5-chamber biofilter (5–6). The water is re-mixed (7) at the entrance to a degassing unit (8–10). After the degasser, the water then enters a 7,300 m^3^ fish tank (11) and is split between an inner and outer raceway ring. The water exits in the fish tanks (12) and re-enters the water treatment loop *via* the drum filters (13). RAS, recirculating aquaculture systems.

The study was performed in one of the two separate RAS modules at Fredrikstad Seafoods (Nordic Aquafarms, Fredriksstad, Norway). The total annual production capacity of these two modules was 1,500 metric tons of 3.5–6 kg Atlantic salmon. Each RAS module has a total water volume of 7,300 m^3^ each, with two circular raceways (1 and 2) for holding fish, Figure 1. Raceway 1 was 4.5 m x 4.7 m x 75 m (width x depth x length) with a total volume of 1,700 m^3^ and a fish production volume of 1,400 m^3^. Raceway 2 was 6.5 m x 4.7 m x 115 m (width x depth x length) with a total water volume of 3,300 m^3^ and a fish production volume of 3,200 m^3^. The two raceways shared the same water treatment with common water in- and out-lets. There was no other mixing of water between the raceways. In addition to the raceways, each RAS module consisted of 6 drum filters of different mesh sizes (ranging from 20 to 50 μm), a moving bed biofilter reactor with 5 chambers, and a degassing unit (Figure 1). The water entered raceways 1 and 2 (point 11, Figure 1) from the water treatment, where it was circulated at speed of 2 and 4 ms^–1^, respectively. The hydraulic retention time (HRT) was 40 min in each raceway. From the outlet of raceways 1 and 2, the water entered the water treatment by flowing into the drum filters (point 13, Figure 1). Thereafter the permeate water was split and flowed either directly into the biofilters (90% of flow) or the biofilter *via* a UV contact chamber (10% of flow). After the biofilter, the water entered the degasser, and then it was pumped back into the raceways. The HRT in the water treatment loop was 22 min. The inflow of new water in the system (make-up water) was 30 m^3^ h^–1^. Fish were kept in continuous artificial light and continuously fed with Bio Mar orbit X feed. The feeding ratio was 0.5% biomass day^–1^ in raceway 1 with newly inserted smolt. Raceway 2 was divided into two compartments up and down streams each other. Fish were fed 1% biomass day^–1^ in one of the compartments. In the other compartment, fish were starved due to slaughter pretreatment. Moreover, in raceway 2, the average weight and number of fish in the compartment containing fed fish that were fed was 4.3 kg and 30,300 fish. The average weight and number of fish in the compartment where fish were pretreated for slaughter were 6.3 kg and 9,500–8,200 fish. The estimated rearing density was about 65 kg m^–3^ in both of these compartments.

### Smolt Insertion and Water Sampling

Smolt insertion was done in raceway 1 during the period of 27–29 of April. Before smolt insertion, the smolts were transported on a truck for approximately 3.5 h. Ten truckloads were inserted over 3 days between 10:00 and 22:00 each day. The smolts had an average weight of 180 g at arrival and were inserted in 850 m^3^ compartment of raceway 1, resulting in a rearing density of 33 kg m^–3^ at the end of the smolt insertion. The reminding of this raceway was kept empty during the period of the study.

Triplicate 0.5 L water grab samples were withdrawn at the inlet and outlet of the raceways. In addition, grab samples collected in the water were sampled in the start, middle, and end sections of the raceways (Figure 1). Water was sampled between 07:00 and 07:30 at depth of 2 m, before the daily husbandry routines started, the day before (April 26), the day after (April 30), and 3 days after (May 2) smolt insertion. The water samples were frozen and kept at −20*^o^*C before being analyzed.

### Water Cortisol Analysis

Analysis of cortisol followed a method described by McWhinney et al. (2010) with some modifications. Water samples were spiked with 10 ng internal standard (cortisol d4) to correct for matrix effects and for losses in sample extraction, concentration, and analysis. Samples were loaded onto activated Oasis HLB 6 cc (200 mg) solid-phase extraction cartridges (Waters, Milford, MA, United States). After loading 200–300 mL of the samples, the columns were washed with 3 ml milliQ water followed by 3 ml of 20% methanol. Samples were eluted with 5 ml 100% ethyl acetate, dried at 50°C, and reconstituted in 200 μL of 40% methanol with 5 mmol/L ammonium formate and 0.1% formic acid. Separation was achieved on a BEH C8 column (Waters, Milford, MA, United States) using a solvent gradient consisting of 5 mmol/L ammonium formate and 0.1% formic acid in water and methanol. Cortisol content was analyzed with a tandem mass spectrometer (Waters TQ-S, Milford, MA, United States) operated in negative electron spray ionization mode with the following MRM acquisition parameters (precursor and product ions); 407.1 > 331.05, 407.1 > 331.1 for cortisol and 411.1 > 335.05, 411.1 > 335.1 for cortisol d4 (IS).

Quantification was done using response factors (Cortisol/Cis) calculated by a 6-point calibration curve from 0.05 to 10 ng/ml. Samples were analyzed in groups with at least one standard addition sample and a blank seawater control. Recovery of spiked cortisol was typically 95–105%. The limit of detection (LOD) of 0.1 ng/L cortisol was estimated as three times the signal to noise (S/N) using spiked control samples.

### Cortisol Production Rate, Clearance Rate, Release Rate, and Clearance Efficacy

For estimation of cortisol clearance rate (CCR; mg cortisol h^–1^) and cortisol release rate (CRR; ng cortisol g fish biomass^–1^ h^–1^), a quasi-steady-state in cortisol was assumed (Good et al., 2014).

Cortisol clearance rate was estimated by multiplying the difference between mean cortisol concentration at the inlet to water treatment [$\bar{\text{x}}$ (cortisol _water treatment_); green in Figure 1] and the cortisol concentration in the three samples withdrawn at the inlet to the raceway [(cortisol _*inlet*_); yellow in Figure 1] with the total volume of the water in the water treatment (V _*treatment*_; total volume-production volume). This was then divided by the HRT of the water treatment.


$$\mathrm{CCR}=\frac{\left(\left[\bar{\text{x}}\,{\mathrm{Cortisol}}_{\mathrm{water}\,\mathrm{treatment}}\right]-\left[{\mathrm{Cotisol}}_{\mathrm{inlet}}\right]\right)\times V_{\mathrm{water}\,\mathrm{treatment}}}{{\mathrm{HRT}}_{\mathrm{water}\,\mathrm{treatment}}}$$

Cortisol release rate in each raceway (CRR_*raceway*_) was estimated by multiplying the difference between the mean inlet concentration [$\bar{\text{x}}$_(*cortisol inlet*)_; yellow in Figure 1] and concentration of the three samples withdrawn in the production volume of the raceway [(cortisol start), (cortisol mid), and (cortisol end); red in Figure 1] with the volume of the raceway (V_*raceway*_). This was then divided by the biomass in the raceway (W_*raceway*_, Table 1) and the hydraulic retention time (HRT_*raceway*_) of the raceway.


$$C\,R\,R_{r\,a\,c\,e\,w\,a\,y}=\frac{\left(\bar{\text{x}}\,\left[C\,o\,r\,t\,i\,s\,o\,l_{i\,n\,l\,e\,t}\right]-\left[C\,o\,r\,t\,i\,s\,o\,l_{r\,a\,c\,e\,w\,a\,y}\right]\right)\times V_{r\,a\,c\,e\,w\,a\,y}}{W_{r\,a\,c\,e\,w\,a\,y}\times H\,R\,T_{r\,a\,c\,e\,w\,a\,y}}$$

**TABLE 1:** Biomass present in the commercial RAS 1 day before, the day after, and 3 days after smolt insertion.

	Biomass (tons)	
	Raceway 1	Raceway 2
Day before smolt insertion	0	191
Day after smolt insertion	28	214
Three days after smolt insertion	28	231

Moreover, cortisol clearance efficiencies were calculated based on the differences between the mean total cortisol production (sum of cortisol production in raceways 1 and 2; mg h^–1^) and mean CCR. Efficiencies were expressed as a percent.

### Feeding Behavior

A husbandry practice response to feeding was routinely quantified by hand-feeding 3–9 times a day. All personal scoring behaviors were trained to rank behavior by the following scale:

1. Non to low locomotor activity when feeding and non to a few fish responds to feeding.
2. Fish responded with a low locomotor activity to hand-feeding and most of the fish do not eat.
3. Fish responded with a medium locomotor activity to hand-feeding and most of the observed fish eats.
4. Fish responded with a high locomotor activity to hand-feeding: all observed fish eats.
5. Fish responded with an even higher locomotor activity to hand-feeding and all observed fish eats.
6. Fish responded with high intensity to hand-feeding, aggression, and all fish eats.

In this feeding score system, feeding score 4 was considered normal feeding behavior.

Because fish were starved before harvested in one of the two rearing compartments in raceway 2, feeding behavior was quantified in the rearing compartment where fish were fed.

### Statistical Analyses

Data from one of the two RAS modules are illustrated in Figure 1. To include individual variances between samples withdrawn in the start, middle, and end sections of the raceways in the analysis, effects of smolt insertion on water cortisol concentrations and CRR in raceways 1 and 2 were analyzed with repeated measure ANOVAs with sampling date as independent variables and sampling place in the raceway as dependent variables.

Effects of smolt insertion on water cortisol concentrations at the inlet to the fish keeping raceways, the inlet to the water treatment, and cortisol removal, were analyzed by ANOVA with sampling date as independent variables. The ANOVA was followed by a Tukey-HSD *post-hoc* test when required. Feeding behavior was analyzed using Kruskal-Wallis tests with sampling dates as an independent variable, followed by Mann-Whitney U tests for testing differences between dates. The values of *p* from the Mann-Whitney U test were corrected for multiple comparisons by Bonferroni correction (α/n), resulting in corrected values of *p* for significant differences (*p* < 0.05) in the inner raceway *p* < 0.01 and the outer raceway *p* < 0.008.

## Results

### Water Cortisol

The cortisol concentrations were changed significantly over time in raceway 1 [ANOVA: *F*_(2,6)_ = 165, *p* < 0.001] and increased significantly compared to values before smolt insertion (*p* < 0.001). Moreover, this increase was highest the day after smolt insertion, showing significantly higher values compared to water sampled 3 d after smolt insertion (*p* < 0.05), Table 2. In addition, cortisol concentrations changed significantly over time in raceway 2, the inlet of fish keeping raceways and the inlet to water treatment [ANOVAs: *F*_(2,4)_ = 290, *p* < 0.001; *F*_(2,6)_ = 118, *p* < 0.001; *F*_(2,6)_ = 50, *p* < 0.001; respectively]. At all these sampling locations, cortisol values were significantly higher after 1 day and 3 days of smolt insertion, compared to the start (*p* < 0.001). However, there were no significant differences in water cortisol concentrations between samples taken after 1 day and 3 days of smolt insertion at raceway 2 (*p* < 0.15), the inlet to the fish keeping raceways (*p* < 0.29), and the inlet to the water treatment (*p* < 0.94).

**TABLE 2:** Concentrations of cortisol in the rearing water in a commercial RAS for slaughter size salmon the day before, the day after, and 3 days after a smolt insertion period of 3 days.

	Water cortisol (ng l^–1^)		
	Day before smolt insertion (26 April)	Day after smolt insertion (30 April)	Three days after smolt insertion (2 May)
In to raceways	0.37 ± 0.01^a^	0.87 ± 0.03^b^	0.81 ± 0.06^b^
Raceway 1	0.37 ± 0.03^a^	1.6 ± 0.1^b^	1.3 ± 0.08^c^
Raceway 2	0.44 ± 0.03^a^	0.98 ± 0.02^b^	1.1 ± 0.06^b^
To water treatment	0.45 ± 0.01^a^	1.1 ± 0.08^b^	1.1 ± 0.06^b^

*Smolts were inserted in raceway 1. This raceway was empty before smolt insertion. Values are averages of three samples ± SEM. Different letters indicate significant differences (p < 0.05) between sampling occasions at each sampling point.*

### Cortisol Release Rate

**FIGURE 2:**
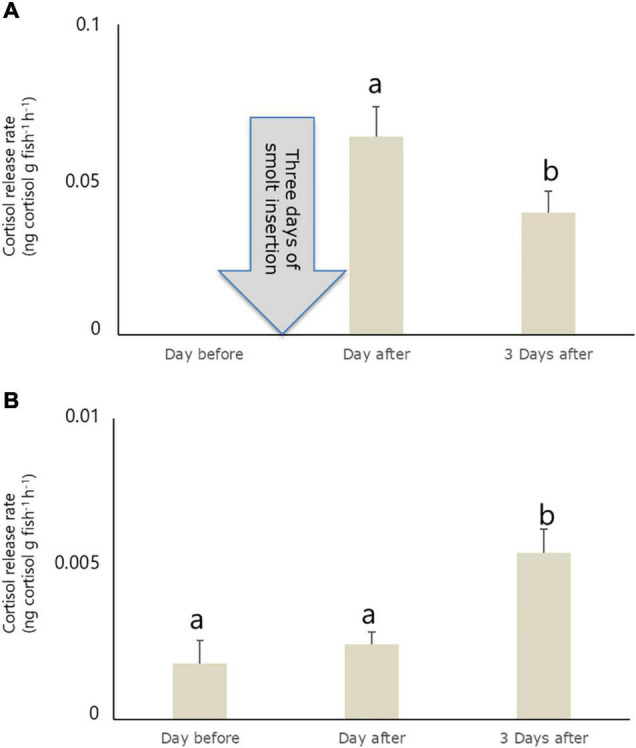
Cortisol release rate in raceway 1 **(A)** and raceway 2 **(B)** before and after a 4 days smolt insertion period in raceway 1. Raceway 1 was emptied before smolt insertion. Different lowercase letters indicate a significant difference (*p* < 0.05) between sampling occasions within a raceway. Values are averages of three samples ± SEM.

Cortisol release rate from fish in raceway 1 decreased significantly between the day after and after 3 days of smolt insertion [ANOVA: *F*_(43)_, *p* < 0.05; Figure 2A]. Also in raceway 2, the CRR changed over time [ANOVA: *F*_(2,4)_ = 8.4, *p* < 0.01], leading to significantly higher values after 3 days of smolt insertion compared to values before *p* < 0.05) and after (*p* < 0.05) smolt insertion. However, CRR 1 day after smolt insertion did not differ significantly from values before smolt insertion (*p* < 0.63) in this raceway (Figure 2B).

### Cortisol Clearance Rate and Clearance Efficacy

Cortisol clearance rates were 0.6 ± 0.1 the day before, 2.0 ± 0.2 the day after, and 2.2 ± 0.24 (mg h^–1^) 3 days after smolt insertion (values are mean ± SE).

Cortisol clearance efficacies were 99.8% the day before smolt insertion, 39.1% the day after smolt insertion, and 43% 3 days after smolt insertion.

### Feeding Behavior

**FIGURE 3:**
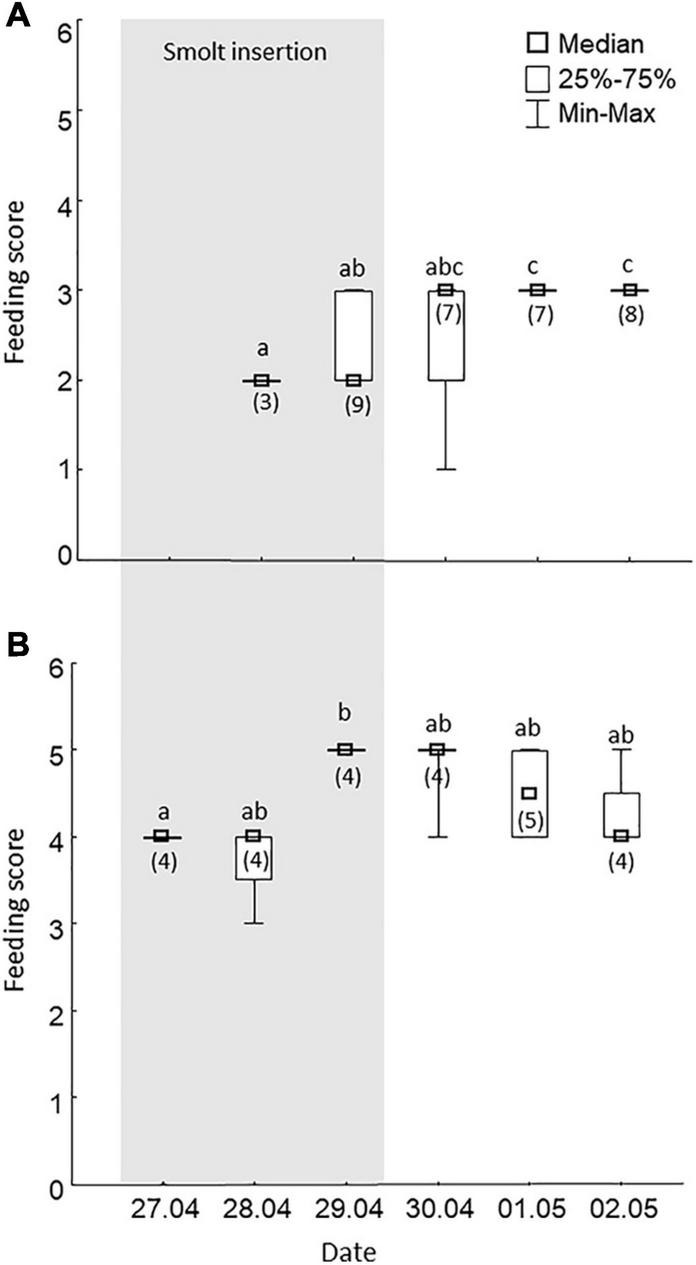
Behavioral response to feeding in Atlantic salmon reared in two raceways in a commercial RAS for slaughter size salmon during a 3-days smolt insertion period (April 27–29) and the three following days (30 April–2 May). Smolts were inserted in raceway 1 **(A)**. This raceway was empty before smolt insertion. Salmon that were established in the system were kept in raceway 2 **(B)**. A feed score of four corresponds to all pellets eaten without observations of aggressive behavior. The feeding score under four score corresponds to that not all pellets are eaten, and the feeding score above four corresponds to that all pellets are eaten and aggressive behavior is observed. More details about the feeding score are shown in section “Materials and Methods.” Numbers within parenthesis denotes the number of daily observations. Different lowercase letters indicate significant differences (*p* = 0.05) between daily feeding scores within a raceway. RAS, recirculating aquaculture systems.

In raceway 1, the feeding score changed over time [Kruskal-Wallis test; H (4) = 16, *p* < 0.003]. This was reflected in significant differences in lower values (*p* < 0.01) scored during smolt insertion (April 28–29) compared to values scored in the two last days following smolt insertion (April 30–May 2; Figure 3). The feeding score also differed significantly between days in raceway 2 [Kruskal-Wallis test; H (5) = 15, *p* < 0.01]. Moreover, in this raceway, the feeding score was significantly higher (*p* < 0.008) on the third day of smolt insertion (April 29) when compared to feeding behavior scored on the first day of smolt insertion (April 27).

## Discussion

In the present study, the water cortisol stayed high for 3 days after smolt insertion in a commercial grow-out RAS for salmon. Moreover, the estimated CRR was approximately ten times higher in newly inserted smolts compared to already stocked fish. Interestingly, salmon acclimatizing to experimental tanks show a similar CRR, ranging from 0.03 to 0.2 ng g^–1^h^–1^ 1–7 d after being inserted in the tanks, as in the current study (Ellis et al., 2007). Underlaying factors for such relative slow adaption have been associated to reduced space, unfamiliar physical environment, and changed social environment (Pottinger and Pickering, 1992). Generally, the transfer to seawater in commercial production consists of several potential stressors, such as capture, loading, transport, unloading, adaption to seawater, new environment, and social structures. In addition, generally, an elevation of plasma cortisol is associated with the process of smoltification (McCormick et al., 2000; Ebbesson et al., 2008). Thus, this suggests that the smoltification process contributed to the increased level of waterborne cortisol in the current study. Still, decreased CRR after smolt insertion in raceway 1 indicates ongoing adaptive processes in the newly inserted smolts.

Mota et al. (2014) estimated cortisol clearance efficacy to be <99% in commercial RAS during baseline conditions. Still, studies comparing steroid concentrations in the make-up water (new water into the system) with concentrations in the rearing water have shown that waterborne cortisol and other steroids accumulate in the rearing water of commercial and experimental RAS (Mota et al., 2014; Good et al., 2017). In the present study, cortisol clearance efficacy was 99.8% 1 day before smolt insertion. However, clearance efficacy decreased to approximately 40% the day after and 3 days after smolt insertion, when cortisol release was increased in the raceways. This demonstrates that the increased water cortisol levels exceeded the cortisol clearance capacity of the system, and contributed to the water cortisol concentrations stayed high after smolt insertion.

A characteristic behavioral response to stress in all vertebrates appears to be a reduction in food intake (Carr, 2002). This is reflected in laboratory studies showing that salmon can respond with a stress-induced anorexia to a new environment (Vaz-Serrano et al., 2011) and resume normal feeding behavior within a week. In accordance with this, the feeding motivation score in the current study raised from score 2 to 3 indicated that the smolts were in the process of adapting to the rearing environment 3 days after being inserted in the raceway. This, together with a reduced water cortisol concentration and release rate 3 days after smolt insertion in raceway 1, suggests that smolts are in the process of adapting to highly intensive seawater rearing in a commercial RAS facility. “However, it is important to note that both feeding score and CRR suggest that fish was not fully adapted during the timespan of the study. Thus, further studies are needed to investigate the time smolt need to fully adapt in a highly intensive RAS.”

Since steroids induce behavioral and physiological changes in fish by acting as pheromones, concerns regarding the behavioral and physiological effects of waterborne steroids in RAS have been raised (Mota et al., 2014; Good and Davidson, 2016; Good et al., 2017). However, these effects seems mainly to be induced by the sex steroids, and whether waterborne cortisol affects fish, has been questioned (Mota et al., 2017b). Interestingly, there was a small increase in cortisol release on day three in raceway 2, containing fish established in the system. However, the current experimental setup did not allow the investigations of whether, and to what extent, this rise in cortisol release was induced by water-borne cortisol. Furthermore, the fish in one of the compartments in raceway 2 were pre-treated for slaughter, such as starvation and handling, and the increased cortisol release may be related to this treatment.

In conclusion, the current results, obtained in a highly intensive commercial RAS, indicate that water cortisol concentrations and CRR are sensitive to system perturbations. Moreover, together with behavioral responses to feed, water cortisol measurements are promising non-invasive indicators of adaptive processes associated with stress resilience in this type of rearing system. As such, they can be used for evaluating the impact of potential stressors, such as compromised water quality, crowding and handling, on fish welfare. However, it is important to keep in mind that cortisol release and clearance rate were estimated assuming a quasi-steady state of hormone release during a smolt insertion event, while the results show a highly dynamic response to this perturbation. Other factors, which can contribute to the dynamics of waterborne cortisol in RAS, include changes in clearance capacity of water treatment processes, amount of new water into the system, and hormone concentrations in this make-up water (Good et al., 2017). Altogether, this accentuates the need for further studies on the underlying mechanisms of the dynamics of water cortisol concentrations, to refine waterborne cortisol as a non-invasive fish-based welfare indicator in commercial RAS.

## Data Availability Statement

The original contributions presented in the study are included in the article/Supplementary Material, further inquiries can be directed to the corresponding author.

## Ethics Statement

We used non-invasive welfare indicators (waterborne cortisol and feeding behavior) to study the impact of smolt insertion in a commercial RAS. All fish treatments in the study are parts of the commercial rearing practice.

## Author Contributions

EH contributed to plan the experiment, draft the manuscript, and data analyses. PF contributed to draft the manuscript and data analyses. PR-T and O-KH-E drafted the manuscript. JR contributed to plan the experiment and data analyses. All authors contributed to the article and approved the submitted version.

## Conflict of Interest

The authors declare that the research was conducted in the absence of any commercial or financial relationships that could be construed as a potential conflict of interest.

## Publisher’s Note

All claims expressed in this article are solely those of the authors and do not necessarily represent those of their affiliated organizations, or those of the publisher, the editors and the reviewers. Any product that may be evaluated in this article, or claim that may be made by its manufacturer, is not guaranteed or endorsed by the publisher.
